# Breast problems and associated factors among lactating women in Northeast Ethiopia, 2022

**DOI:** 10.1038/s41598-024-58957-0

**Published:** 2024-04-22

**Authors:** Anguach Shitie, Abebe Adimasu, Delelegn Tsegaye, Dagne Belete, Esuyawkal Mislu, Mandefro Assfaw, Wondimnew Gashaw Kettema

**Affiliations:** 1 College of Medicine and Health Sciences, Wollo University, Dessie, Ethiopia; 2 College of Health Sciences, Woldia University, Woldia, Ethiopia

**Keywords:** Breast problems, Lactating women, Breast health, Lactation, Health care, Public health, Infectious diseases

## Abstract

Breastfeeding is the cornerstone of child and maternal health. However, maternal breast problems during breastfeeding have been frequently reported as one of the reasons for early discontinuation of breastfeeding. Despite the importance of having knowledge on breast problems magnitude and its associated factors in the clinical practices and designing effective interventions, there is limited data on this topic. Therefore, this study aimed to assess the prevalence and associated factors of breast problem among postnatal lactating women in Legambo district, south wollo zone, North East Ethiopia, in 2022. A community-based cross sectional study was conducted among 610 lactating mothers in Legambo district. Multi-stage sampling was employed to select study participants. Interviewer administered, WHO B-R-E-A-S-T-Feeding, observational checklist and maternal self-reported breast problem questionnaires were used to collect the data. Epi-Data version 3.1 was used for data entry and export to SPSS version 25.0 for analysis. Descriptive statistics and bi-variable and multivariable analysis was carried out. On the multivariable logistic regression, variables with p-value < 0.5 were considered as they had statistically significant association with breast problem. The overall prevalence of breast problems among postnatal lactating women was 54.3% (95%, CI 49.3–59.3%). Primipara (AOR = 5.09; 95% CI 3.40–7.62), preterm infant (AOR = 2.12; 95% CI 1.22–3.66), home delivery (AOR = 3.67; 95% CI 1.62–8.30), ineffective breastfeeding techniques (AOR = 2.45; 95%CI 1.61–3.74), caesarean section delivery (AOR = 2.05;95%CI :1.15–3.64) and mixed type of feeding (AOR = 1.97:95%CI 1.34–2.89) were factors showed significant association. The prevalence of breast problems was 54.3%. Relevant factors related to an increase risks are being primipara, cesarean section delivery, home delivery, preterm birth, ineffective breastfeeding techniques and mixed type of feeding.

## Introduction

Breastfeeding and weaning, unlike in other mammalian species, are not solely directed by nature for human beings. As a result, the process of breastfeeding and weaning must be taught. In modern societies, women have limited opportunities to learn about breastfeeding, primarily due to the loss of traditional sources of knowledge, such as experienced women in extended families, as nuclear families have become more prevalent^1^. Unfortunately, improper breastfeeding practices and the use of supplemental or mixed feeding have become increasingly common worldwide^2^. Breastfeeding is essential for the survival, nutrition, development of children and maternal health^3^.

To ensure sufficient milk production and flow for 6 months of exclusive breastfeeding, proper support should be provided^4^. Maternal breast problems during breastfeeding are often reported as one of the reasons for early discontinuation of breastfeeding^5–9^. The postnatal period is a critical phase in the lives of mothers and newborn babies, with a higher risk of maternal and infant mortality. However, this period is often neglected in terms of quality care provision^10^. Non-breastfed children are more susceptible to severe cases of diarrhea^3^ and pneumonia^1^ which are leading causes of death among infants.

Breast problems like breast engorgement, flat or inverted nipples, cracked nipples, mastitis, and abscess affects many lactating women, particularly those who did not practice effective exclusive breastfeeding in the postpartum period. If not managed properly and in a timely manner, these problems may escalate and increase the risk of breast cancer, which is one of the leading causes of death for women^11^. Breast problems often remain hidden with in communities^12,13^. It is now clear that addressing breast problems contributes to the overall benefits of breastfeeding practices for infants, mothers, families, society, and the country as a whole^14,15^.

Despite the importance of understanding the magnitude of breast problems and their associated factors for effective clinical practices and intervention design, there is a scarcity of evidence at both the local and national levels. Therefore, this study aimed to assess the prevalence and associated factors of breast problems among postnatal lactating women in Legambo District, Northeast Ethiopia. The findings of this study will play a crucial role in designing prevention and management policies and strategies, as well as improving the quality of services related to breast problems.

## Methods

### Study area and period

Community based cross sectional study was conducted in Legambo District from September 1–September 30, 2022. The district is located 100 km from Dessie, and 601 km from Addis Ababa (the capital city of Ethiopia). It has 41 kebeles, of which 38 are rural kebeles and three are urban kebeles. The district has 9 health centers, 39 health posts, and a single hospital. The 2022 statistical figure of Legambo District Health Bureau report showed that, the total population in the district was around 364,452, of which 172,748 are females^16^.

### Source population

All lactating women living in Legambo district were our source population.

### Study population

The study population was lactating women who give birth in the last 6 weeks and living in selected kebeles.

### Eligibility criteria

All postnatal women who breastfeed a child at the time of study period were included in to the study. Mother whose infant were died or seriously ill, unable to suck, lethargic, or unconscious were excluded from the study.

### Sample size determination

A single population proportion formula was used to calculate the sample size for this study with the assumption of a 95% confidence interval (CI), a margin of error of 5% and a 50% proportion.

$$ {\text{Sample size }} = \, \left[ {\left( {{\text{Z1}} - \upalpha /{2}} \right)^{{2}} \cdot {\text{ P }}\left( {{1} - {\text{P}}} \right)} \right]/{\text{d}}^{{2}} $$

where,

(Z1-α/2) = is standard normal variation, P = proportion in the population of our study area, we used 50% because there was no previous study with similar study population, d = absolute error or precision, Sample size = [(1.96)^2^. (0.50). (1–0.5)]/(0.05)^2^ = 384.

Multi-stage was employed for sampling methods from district to kebeles and kebeles to house hold level, so sample size would be multiplied by 1.5, n = 384 * 1.5 = 576.

Considering 10% non-response rate, the total sample size was determined to be 634 postnatal mothers.

### Sampling procedure

A multi-stage sampling method was used to select a representative sample. In the first stage, a simple random sampling technique was used to select eighteen kebeles from forty-one kebeles. From 38 rural kebeles, 16 kebeles were selected, and from the urban three kebeles, two kebeles were selected by simple random sampling. The number of women who delivered in each selected kebele was taken from the health extension worker's registration book. Then proportionally allocate the sample to each selected kebele. Finally, study participants were selected by simple random sampling by using the list of women in the extension worker registration book as a frame.

### Variables

#### Dependent variable

Breast problem.

#### Independent variables

*Socio-demographic* age, educational status, residence, marital status and occupation of the mother.

*Obstetric characteristics* parity, place of delivery, mode of delivery, history of breast problems, gestational age at delivery, antenatal care follow up, postnatal care follow up,

*Breastfeeding characteristics* time of breastfeeding initiation, positioning, attachment, suckling, prelactal feeding, breastfeeding technique and additional type of feeding given with breast milk, knowledge on benefits of breastfeeding, previous feeding experience and source of information about breastfeeding.

#### Operational definition

*Breast problems* if a woman has any one or more of the following six conditions:

*Inverted nipple* a portion of or the entire nipple is buried below the plane of the areola^17^.

*Flat nipples* nipples part at the plane of the areola^3^.

*Crackle* damage to the integrity of the skin of nipple^18^.

*Engorgement* is swelling and distension of the breasts, usually in the early days of initiation of lactation, caused by vascular dilation as well as the arrival of the early milk, and the women may experience extreme fullness, tightness, and pain in the breasts. Additionally, the skin may appear shiny and stretched, and the breasts may feel warm to the touch^19^.

*Mastitis* is a tender, hot, swollen, wedge-shaped area of breast associated Fever (38 Degree Celsius), chills, flu-like aching, and systemic illness^20^.

*Breast abscess *is a painful fluctuant swelling in the breast, which feels full of fluid, may be discoloration of the skin at the point of the swelling^4^.

##### Effective breastfeeding technique

The achievement of the combination of at least two criteria from positioning, three criteria from the attachment, and two criteria from suckling while mothers' breastfeeds their infant^10^.

##### Poor, average and good positioning

Women fulfill none of or only one, any two and all the four/three out of four criteria will be considered respectively^17^.

##### Poor, average and good attachment

Women fulfill none of or only one criteria, any two of the criteria and all four/three criteria out of four criteria for infant attachment will be considered respectively^3^.

##### Poor suckling

None of or only one of the three criteria have been achieve^21^.

##### Knowledge on benefits of breastfeeding

Women who correctly answers any seven or more out of thirteen questions was consider as having good knowledge about benefits of breastfeeding^22^.

### Data collection tools and procedure

The data were collected by using interviewer-administered questionnaires, an observational checklist, and maternal self-reported signs and symptoms of breast problems. The questionnaires included socio-demographic, obstetrical, breastfeeding, maternal healthcare, and breast problem-related characteristics. An observational checklist, based on the World Health Organization (WHO) B-R-E-A-S-T-Feeding observation form^23^, was used to assess mother and baby’s position; attachment, and suckling during breastfeeding. This form was developed by the WHO and United Nations Children’s Fund (UNICEF) to suit for low resource settings^24^. The tool were developed and modified from relevant studies^22,25–28^. Interviewer-administered questionnaires were initially prepared in English and then translated into the local language (Amharic) by language experts.

To evaluate positioning, attachment, and suckling during breastfeeding, an arbitrary scoring and grading system was adopted and developed based on WHO criteria^12^, with each criterion assigned a value (Table 1).

**Table 1:** Socio-demographic characteristics of the participants for the study conducted in Legambo district, north east Ethiopia, 2022 (n = 610).

Variables	Frequency	%
Age of the mothers		
˂ 20	67	11.0
20–25	278	45.6
26–30	162	26.6
> 30	103	16.9
Residence		
Rural	327	53.6
Urban	283	46.4
Level of education		
No formal education	266	43.6
Primary education	229	37.7
Secondary education	80	13.1
More than secondary	35	5.7
Marital status		
Single	54	8.9
Married	545	89.3
Others^a^	11	1.8
Occupation		
House wife	471	77.2
Working	139	22.8

Others^a^: widowed and divorced.

Ten trained female midwives with Bachelor's degrees (BSc.) collected the data after receiving training through video demonstrations and hands-on practice. The data collectors observed breastfeeding practices for four minutes and recorded the positioning, attachment, and suckling using the WHO B-R-E-A-S-T-Feeding observation checklist form^23^. Postnatal lactating mothers were approached, their willingness to participate in the study was assessed, and interviews and observations were conducted only after obtaining written consent.

### Data quality control

To assure data quality, both the data collectors and supervisors received a 2-day training covering the study’s objectives, relevance, expected challenges and difficulties, confidentiality of information, respondents’ rights, informed consent, and interview techniques. A pretest was conducted in Dessie Zuria involving 5% (32 women) of the total sample size, before the actual data collection period. This was aimed to assess the instrument simplicity, flow, and consistency, as well as to check the instrument’s reliability. The overall reliability, measured by Cronbach alpha, was found to be 0.78. Each questionnaire was checked for completeness and unlikely responses prior to data entry. The data were then edited, coded, and entered into Epi Data version 3.1. Two data clerks performed double data entry, and entry errors were checked and corrected by referring back to the questionnaires.

### Data processing and analysis

Data clerks and the supervisor visually checked for completeness before being coded and entered into the computer using Epi-Data version 3.1. During the analysis process using SPSS version 25.0, descriptive statistics were performed on each variable, and the results were presented in tables, figures, and texts using summary measures such as percentage, mean, and standard deviation.

Bivariable logistic regression was conducted, and variables with p-values less than 0.25 were included in the multivariable logistic regression to examine the relative effect of the confounding variable and variable interaction. Variables with a p-value of 0.05 were considered statistically significant in multivariable logistic regression analysis. The omnibus test of model coefficients and Hosmer and Lemeshow goodness-of-fit test were used to assess the overall model summary and model goodness of fit, respectively, yielding values of 0.000 and 0.834, respectively. The variance inflation factor (VIF) and tolerance test were used to check for multicollinearity among the explanatory variables, with the highest VIF being 1.16 and the lowest tolerance test being 86%.

### Ethics approval and consent to participate

Ethical clearance was obtained from the Wollo University College of Medicine and Health Sciences Ethical Review Board (Ref.No CMHS/004/13). The study was conducted in adherence to the Declaration of Helsinki and relevant guidelines and regulations. Written informed consent was received from all participants prior to actual data collection.

## Results

### Socio-demographic characteristics of the study participants

From the 634 study participants, 610 lactating mothers were observed for breast problems and interviewed, giving a response rate of 96.2%. About 278 (45.6%) of mothers were in the age group of 20–25 years, and the mean age of the study participants was 25.69 years, with SD** ± **6.014 years. The majority of the respondents were housewives and rural dwellers, 77.2% and 53.6%, respectively. Near to Ninety percent of the respondents (89.3%) were married. Regarding their level of education, 266 (43.6%) had no formal education (Table 1).

### Obstetrics characteristic of the respondents

Of the total participants, 380 (62.3%) were multipara and 56 (9.2%) gave birth at home. Regarding mode of delivery, 517 (84.8%) were give births by spontaneous vaginal deliveries. Of 534 (87.5%), infants were born at term gestational age and 554 (90.8%) of postpartum women had no previous breast problem history (Table 2).

**Table 2:** Obstetrics characteristics of the participants for the study conducted in Legambo district, north east Ethiopia, 2022 (n = 610).

Variables	Frequency	%
Parity		
Primipara	230	37.7
Multipara	380	62.3
Place of birth		
Home	56	9.2
Health institution	554	90.8
Mode of delivery		
SVD	517	84.8
CS	93	15.2
GA in complete weeks		
Preterm	104	17.0
Term	506	83.0
Previous history of breast problems		
Yes	56	9.2
No	554	90.8

*SVD* spontaneous vaginal delivery, *CS* cesarean delivery, *GA* gestational age.

### Breastfeeding characteristics of the study participants

Of the total participants 236 (61.3%) of postnatal lactating mother were give prelacteal feed for their infant and only 74 (12.1%) of postnatal lactating mother were initiated breastfeeding within one hour of delivery. Of the total participants around frothy percent (38.2%) of postnatal lactating mothers mixed feed their infants with other food and fluid in addition to breast milk at the time of the interview. More than seventy four percent (74.18 percentage) of the mother practiced ineffective breastfeeding techniques (Table 3).

**Table 3:** Breastfeeding characteristics of the participants for the study conducted in Legambo district, north east Ethiopia, 2022 (n = 610).

Variables	Frequency	Percent (%)
Prelactal feed		
Yes	236	38.7
No	374	61.3
Time of breastfeeding initiation		
Within 1 h	74	12.1
2–3 h	484	79.3
First day (4–24 h)	49	8.0
At or after 24h	3	0.5
Type of feeding		
Exclusive breastfeeding	377	61.8
Mixed feeding	233	38.2
Breastfeeding technique		
Effective breastfeeding technique	158	25.9
Ineffective breastfeeding technique	452	74.1

### Maternal and health care system characteristic

Around seventy five percent 456 (74.8%) of the study participants had antenatal care (ANC) follow up. More than 80 percent (81.97%) of mothers received postnatal care. Just over fifty five percent 341 (55.9%) of the study participants had received information about breastfeeding from their friends and family. Regarding their breastfeeding experience 295 (48.4%) of postnatal women had previous experience**.** Out of 610 mothers, 346 (56.7%) had good knowledge on the benefits of breastfeeding for maternal and child health (Table 4).

**Table 4:** Maternal and health care characteristics of the study participants for the study conducted in Legambo district, north east Ethiopia, 2022 (n = 610).

Variables	Frequency	%
ANC follow up		
Yes	456	74.8
No	154	25.2
PNC follow-up		
Yes	500	81.97
No	110	18.03
Source of information on breastfeeding		
Friends and family	341	55.9
Health care providers	160	26.2
Audio visual^a^	109	17.9
Previous breastfeeding experience		
Yes	295	48.4
No	315	51.6
Mother knowledge on benefits of BF		
Good knowledge	346	56.7
Poor knowledge	264	43.3

Audio visual^a^-radio, TV, Newspaper and internet.

### Individual breast problem sign and symptoms

The commonest self-reported symptoms of breast problems were pain (55.7%), difficulty of milking or difficulty to feed and attach breast (47.5%) (Table 5).

**Table 5:** Prevalence of individual breast sign and symptoms among postnatal lactating women in Legambo district, Northeast Ethiopia, 2022 (n = 610).

Variables	Frequency	Percent (%)
Damage to integrity of the skin of nipple(crackle nipple)	77	12.6
Portion or the entire nipple buried below the plane of areola(inverted nipple)	30	4.9
Nipple part at the plane of areola (flat nipple)	13	2.1
Engorgement	220	36.1
Swelling	241	39.5
Breast distension	273	44.8
Shiny and lumpy breast	241	39.5
Difficulty of milking	290	47.5
Difficulty to feed and attach	290	47.5
Whole breast affected	260	42.6
Mastitis	61	10.0
Redness of overlaying skin	92	15.1
Lump	143	23.4
Pain	340	55.7
Only part of one breast affected	55	9
Wedge shaped area of breast	54	8.9
Shivering or chills	50	8.2
Headache or body ache	123	20.2
Fever	94	15.4
Abscess	12	2.0
Fluctuant lump	12	2.0
Skin discoloration	12	2.0
Breast feels full of fluid	12	2.0
Overall breast problem	316	54.3

### Prevalence of breast problems

The overall prevalence of breast problems was 54.3% (95%, CI 49.3–59.3%). Among those problems, breast engorgement was the most commonest one, accounting for around thirty six percent (36.1%), followed by crackle nipple, mastitis, inverted nipple, flat nipple, and abscess (12.6%, 10.0%, 4.90%, 2.10%, and 2.00%), respectively.

### Factors associated with breast problems

In the bi-variable logistic regression analysis, eleven variables had p-values less than 0.25 and were entered into multivariable logistic regression (occupation, age of mothers, place of birth, parity, gestational age, mode of delivery, previous breast problem history, pre-lacteal feeding, breastfeeding experience, breastfeeding technique and type of feeding). Home delivery, primipara, preterm infants, cesarean delivery, ineffective breastfeeding technique and mixed type of feeding were identified as having a significantly association with breast problems on the multivariable analysis.

This study identified that the odds of breast problem was 3.67 times (AOR = 3.67; 95% CI 1.62, 8.30) higher among women who give birth at home than health institutions. Similarly, the odds in primipara were 5.09 times (AOR = 5.09; 95% CI 3.40, 7.62) greater compared to multigravida mothers, and 2.12 times (AOR = 2.12; 95% CI 1.22–3.66) more in mother who gave a preterm infant than term infant.

Additionally, the odds of breast problem among women who delivered by caesarean section were 2.45 times (AOR = 2.45; 95% CI 1.61–3.74) higher as compared with vaginal delivery, and the odds was 1.55 times (AOR = 1.55; 95% CI 1.07, 2.25) times higher among women who practice ineffective breastfeeding techniques than effective breastfeeding techniques. Moreover, the odds of breast problem in women who provide mixed feed were 1.97 times (AOR = 1.97:95% CI 1.34, 2.89) higher than women who feed breast milk exclusively (Table 6).

**Table 6:** Bi-variable and multivariable analysis of factors associated with breast problems among postnatal lactating mother in Legambo district, Northeast Ethiopia, 2022(n = 610).

Variables		Breast problems		COR (95%CI)	AOR (95%CI)
		No	Yes		
Occupation	House wife	220 (46.7%)	251 (53.3%	1.3 (0.89, 1.89)	0.67 (0.43–1.05)
	Working	74 (53.3%)	65 (46.8%)	1	1
Age of mothers	≤ 20	33 (11.2%)	34 (10.8%)	0.80 (0.43, 1.48)	0.50 (0.25–1.04)
	20–25	140 (47.6%)	138 (43.7%)	0.77 (0.49, 1.20)	0.85 (0.50–1.44)
	26–30	76 (25.9%)	86 (27.2%)	0.88 (0.53, 1.44)	0.97 (0.55–1.72)
	≥ 30	45 (15.3%)	58 (18.4%)	1	
Place of birth	Home	10 (26.0%)	44 (28.0%)	4.60 (2.27, 9.31)	3.67 (1.62–8.30)*
	health institutions	284 (51.1%)	272 (48.9%)	1	1
Parity	Primi	57 (24.8%)	173 (75.2%)	5.33 (3.50, 7.24)	5.09 (3.40–7.62)**
	Multi	237 (62.4%)	143 (37.6%)	1	1
gestational age	Preterm	29 (18.6%)	75 (81.4%)	2.84 (1.79, 4.52)	2.12 (1.22–3.66)*
	Term	265 (50.2%)	241 (49.8%)	1	1
Delivery mode	SVD	268 (51.8%)	249 (48.2%)	1	1
	CS	26 (28.0%)	66 (72.0%)	2.77 (1.70, 4.50)	2.45 (1.61–3.74)*
breast problem history	Yes	32 (57.1%)	24 (42.9%)	0.67 (0.39, 1.17)	0.719 (0.39–1.35)
	No	262 (47.3%)	292 (52.7%)	1	1
Prelactal feed	Yes	121 (51.3%)	115 (48.7%)	0.82 (0.59, 1.13)	0.95 (0.65–1.38)
	No	173 (46.3%)	201 (53.7%)	1	1
BF experience	Yes	151 (51.2%)	144 (48.8%)	0.79 (0.58, 1.09)	0.97 (0.65–1.43)
	No	143 (45.4%)	172 (54.6%)	1	1
Breastfeeding technique	Effective	132 (55.7%)	105 (44.3%)	1	1
	Ineffective	162 (43.4%)	211 (56.6%)	2.54 (1.75, 3.68)	1.55 (1.07–2.25)**
Feeding type	Mixed	91 (31%)	142 (44.9%)	1.82 (1.30, 2.54)	1.97 (1.34–2.89)**
	EBF	203 (69.0%)	174 (55.1%)	1	1

*Statistically significant p-values < 0.05; **statistically highly significant < 0.001.

## Discussion

This study identified that the prevalence of breast problems in the district during postnatal period was 54.3% (95% CI 49.3–59.3%). Among these problems, breast engorgement was the most common (36.1%), followed by cracked nipple, mastitis, inverted nipple, flat nipple, and abscess (12.6%, 10.0%, 4.90%, 2.10%, and 2.00% respectively). However, a study conducted in Iran showed that the breast fissure as the most common breast problem followed by inverted nipples and mastitis^29^. A study conducted in Egypt reported a prevalence of 82%^30^ and in India it ranges from 65 to 75%^31^, which is higher than the finding of the current study. Conversely, the current study’s results were higher than studies conducted in United States 9.5%^32^, South Asia 8%^25^, India (5.1%)^33^, and Australia 20%^26^. These differences might be due to variations in the study design, inclusion criteria, and sampling methods.

The current study’s findings are in line with studies conducted in Egypt (Tanta and Helwan university) where more than half of the mothers (54.6%) developed postpartum breast problems^16^. This study’s findings are also consistent with a study conducted in Bangladesh, which reported rates of 7.89%, 2.63%, and 1.75%)^2^ for cracked, inverted, and flat nipple respectively. Additionally, the abscess rate in Egypt (Tanta) was (2.75%)^3^, and in Egypt (Zagazig University) it was (1.28%)^15^, which are similar to the findings of this study.

Primiparous women were more likely than multiparous to develop breast problems. This finding is supported by a study conducted in India^3^. The possible explanation for this is that multiparous women have a higher degree of awareness of breast problems in the first 3 days after birth compared to primiparous women. Primigravida women are also at a higher risk of labor complications, which can result in cesarean delivery and a lower likelihood of timely initiation of breastfeeding. Additionally, primiparous mothers may have poorer attachment and positioning practices compared to multiparous women^25^, and multiparous women are more likely to have knowledge of childcare and feeding techniques from their previous child.

Women with preterm infants had higher odds of developing breast problems compared to mothers with term infants. This finding is supported by a study conducted in Texas^27^. This difference might be due to restrictions on mother-infant continuous contact in neonatal intensive care units, as well as delays in discharge and limited contact with the mother after overcoming complications such as intraventricular hemorrhage and necrotizing enterocolitis. Preterm infants may also have difficulty successfully suckling breast milk compared to full-term infants, as supported by a study conducted in Bangladesh^22^. Additionally, the younger age of preterm newborns contributes to their limited physical maturity and physiological functioning, which delays their ability to attach to the breast properly^12^.

Women who delivered by caesarean section were more likely to develop breast problems compared to those who had spontaneous vaginal delivery. This finding is supported by studies conducted in many countries^2,23,34–36^. The possible explanation might be the adverse association between cesarean section and breastfeeding. Post-operative care routines may interrupt the onset of lactation, disturb mother-infant interaction, and inhibit infant suckling. Mothers who undergo cesarean section might be under the effect of anesthesia for extended periods after the operation, which can prevent from regular emptying of the breasts and increase the risk of milk stasis and breast problems. Additionally, the immediate postpartum period is challenging for mothers who have undergone cesarean delivery due to pain, which can affect their ability to breastfeed early and contribute to breast problems.

Women who gave birth at home had higher odds of developing breast problems compared to those who gave birth at a health institution. This finding is supported by a study conducted in Bangladeshi^6^. This might be due to the lack of counseling and support received by women who deliver at home, as they may not have access to breastfeeding, attachment, and positioning guidance. In contrast, women who give birth at health institutions are more likely to receive counseling services and initiate breastfeeding early.

Women who practice ineffective breastfeeding were more likely to develop breast problems than those who practice effective breastfeeding techniques. This finding is supported by a study conducted in Brazil^26^. Poor positioning, attachment, weak or uncoordinated suckling, and incorrect handling of the infant at the mother's breast can lead to nipple trauma and contribute to the development of breast problems. Furthermore, inefficient removal of breast milk can lead to milk stagnation and engorgement, making attachment and suckling more challenging. This can progress to more severe forms of breast problems such as mastitis and abscess^1^.

Women who supplement with mixed foods and fluids for infants were more likely to develop breast problems than those who exclusively breastfed. This might be due to the intake of additional food and liquids reducing the frequency and duration of breastfeeding, leading to inadequate breast emptying and increased risk of breast problems. Moreover, this practice increases the risk for the development of illnesses, such as pneumonia or diarrhea, which affects some aspects of the sucking technique and regular breast milk emptying^3,25^.

This study has certain limitations that should be considered when interpreting the results. Firstly, the use of interviewer-administered questions might introduce recall bias. Additionally, the Hawthorne effect might present while trying to use the observational checklist. However, attempts were made to minimize this effect by concealing the observation from the study participants.

## Conclusion

The study revealed a prevalence rate of 54.3% for breast problems during the postnatal period. Parity, mode of delivery, place of delivery, preterm birth, ineffective breastfeeding techniques, and mixed feeding were identified as significant factors associated with these problems. Given that breast problems can lead to adverse effects like feeding difficulties, decreased milk supply, compromised emotional bonding, and suboptimal weight gain for the child, as well as chronic inflammation and associated non-breastfeeding can also increase future risk of developing breast cancer. Therefore, it is imperative to address, prevent, and manage breast problems effectively, and ensuring access to quality healthcare services with appropriate health professional mix. Health care providers and researchers can also tailor interventions and support strategies accordingly.

## Data Availability

Full data of this research is available upon request from the corresponding author (esuyawkalmislu@gmail.com).

## Abbreviations

AOR: Adjusted odd ratio
COR: Crude odd ratio
CS: Cesarean section
EBT: Effective breastfeeding technique
EDHS: Ethiopian Demographic Health Survey
HEW: Health extension worker
IBT: Ineffective breastfeeding technique
NICU: Neonatal intensive care unit
PNC: Postnatal care
